# Spatiotemporal dynamics of syphilis in pregnant women and congenital syphilis in the state of São Paulo, Brazil

**DOI:** 10.1038/s41598-021-04530-y

**Published:** 2022-01-12

**Authors:** Joelma Alexandra Ruberti Medeiros, Mellina Yamamura, Zilda Pereira da Silva, Carmen Silvia Bruniera Domingues, Eliseu Alves Waldman, Francisco Chiaravalloti-Neto

**Affiliations:** 1 School of Public Health, University of São Paulo, São Paulo, São Paulo Brazil; 2 Nursing Department, Federal University of São Carlos, São Carlos, São Paulo Brazil; 3 Reference and Training Center for Sexually Transmitted Diseases and AIDS, STI/AIDS State Program, Coordination of Disease Control, São Paulo State Department of Health, São Paulo, SP Brazil

**Keywords:** Health care, Public health, Epidemiology

## Abstract

We aimed to estimate the occurrence of syphilis in pregnant women (SPW) and congenital syphilis (CS) in the municipalities of the state of São Paulo (SP) and evaluate their relationship with socioeconomic, demographic, and health care variables. We developed an ecological study based on secondary data of SPW and CS with spatiotemporal components from 645 municipalities in SP including data from 2007 to 2018. We modeled the data in a Bayesian context, considered spatial and temporal random effects, and used binomial negative probability distributions. We found a continuous increase in the relative temporal risk of SPW, from 2007 to 2018, and CS, from 2007 to 2017, when their incidences increased by 8.6 and 6.6 times, respectively. This increase occurred en bloc in practically all municipalities of SP. The increase in SPW was associated with teenage pregnancy, municipalities with a large number of inhabitants, and acquired immunodeficiency syndrome (AIDS) incidence. The increase in CS was associated with municipalities with a large number of inhabitants, incomplete antenatal care, and AIDS incidence. Although actions to control these diseases are required in all municipalities of SP, the identification of high-risk areas points to priority regions for development.

## Introduction

Syphilis is a disease whose etiological agent is *Treponema pallidum*. With the discovery of penicillin, the disease was expected to be eliminated. Nevertheless, the number of infected adults, pregnant women, and children has increased significantly^1,2^. Pregnant women with syphilis who do not receive the correct treatment may experience fetal and neonatal deaths, preterm births, and babies with low birth weight. Live births (LB) can suffer from the clinical effects and consequences of *T. pallidum* infections.

The factors that contribute to the acquisition of syphilis by the women are related to socioeconomic and cultural aspects, such as gender inequalities, low education level, adolescent early sexual activity, and drug use. These factors are complemented by certain biological characteristics of women, such as genital epithelial disruptions and the difficulty to identify the signs and symptoms of syphilis^3^. To manage this scenario, it is necessary for women to have access to health care and adequate treatment; this highlights the role of primary health care (PHC) to deal with the diagnosis, notification, surveillance, and treatment of syphilis.

In 2006, the Pan American Health Organization and other organizations agreed on the targets for the elimination of congenital syphilis (CS)^4^. However, even with the implementation of these initiatives, CS and syphilis in pregnant women (SPW) have remained increasingly serious public health problems^1,2^. Korenromp et al.^5^ estimated that in 2016, the global prevalence of SPW was 0.69% and the global occurrence of CS was 661,000 cases. Accordingly, 355,000 cases with adverse outcomes were estimated, with 143,000 early fetal deaths and stillbirths, and 61,000 neonatal deaths. They also estimated the occurrence of 41,000 and 109,000 preterm or low-birth-weight births and children with clinical illnesses, respectively^5^. To deal with this picture, the Pan American Health Organization recently developed a specific model for transmissible diseases, including acquired syphilis (AS), SPW, and CS, that articulates directly to the sustainable development goal number 3 of the 2030 Agenda^6^.

In Brazil, the detection rate of AS has increased significantly in all age groups, especially in those aged 20–29 years. Between 2014 and 2018, its detection rate increased from 2.1 to 75.8 cases per 100,000 inhabitants. The SPW detection rates, between 2008 and 2018, evolved from 2.5 to 21.4 cases per 1000 LB. The CS incidence rate increased from 1.9 cases per 1000 LB in 2007 to 9.0, in 2018. The state of São Paulo (SP) had similar rates. A comparison of the years 2007 and 2018 showed that the detection rate of SPW increased by 11.6 times (from 1.8 to 20.9 cases per 1000 LB) and the incidence rate of CS increased by 5.1 times (from 1.3 to 6.6 cases per 1000 LB)^7^.

Important aspects in the study of syphilis, especially SPW and CS, are the evaluations of their spatial distributions and temporal evolutions, which are generally made using ecological designs. These evaluations allow the visualization of places with a greater probability of occurrence, association of occurrence with the conditions of the territories with populations at risk, identification of risk areas that need prioritization, and assessment of the effectiveness of surveillance and control measures^8^. Furthermore, the most adequate design when we considering space and time are the models with a space–time architecture. They allow these two dimensions to be considered simultaneously and the relationship with possible associated factors to be adjusted for the spatial and temporal autocorrelations of the response variables^9,10^.

However, few ecological studies with spatial or space–time designs for investigating SPW and CS have been conducted in Brazil. We conducted a search on PubMed, Web of Science, and Scopus for studies published from 2010 to 2020 and found only four studies conducted in Brazil on this theme^11–14^, highlighting the existence of a knowledge gap as none of the studies used space–time modeling, few focused on low-income countries, and none used SP as a study area. Thus, we aimed to estimate the occurrence of SPW and CS in the municipalities of SP and evaluate their relationships with socioeconomic, demographic, and health care variables.

## Results

In SP, between 2007 and 2018, the Information System for Notifiable Diseases (SINAN) received notifications of 54,844 cases of SPW and 27,729 cases of CS. This corresponded to a global incidence rate of 7.50 SPW cases per 1000 LB-year and a global incidence rate of 3.79 CS cases per 1000 LB-year, considering that there were 7,313,551 LB during the study period. The expected values of the SPW and CS cases in each municipality and year were calculated using these rates. While the number of LB increased by only 1.8% from 2007 to 2018, the number of notified cases of SPW and CS increased 10.8 and 4.1 times, respectively.

We performed a descriptive analysis of the characteristics of women with gestational syphilis and CS, using and comparing the data from 2007 and 2018, respectively, which is presented in Supplementary Material 1. The majority of SPW and CS cases were associated with women aged between 20 and 29 years old, with 5 and 12 years of education. In 2007, most women declared themselves white; however this changed in 2018 when most women declared either black or brown. Around two-thirds or more of the SPW cases were diagnosed during the first or second trimester of pregnancy, with more than 80.0% mothers receiving adequate treatment from approximately 60.0% of the municipalities. In CS cases, more than 80.0% mothers received prenatal care, but only 57.0% were diagnosed during the prenatal period; less than 7.2% underwent adequate treatment against syphilis. Additionally, less than 23.1% of these mothers' partners were treated for the disease. It is noteworthy that some of these variables presented high percentages of ignored information; highlighting up to 33.3% missing data on schooling and up to 20.5% on partner’s treatment.

In the database (Supplementary Material 2), there were null values in 52.0% of the data for SPW and 64.6% for CS; hence, zero-inflated probability distributions were considered. The models with Poisson probability distribution, both non-inflated and zero-inflated models, showed an overdispersion and were disregarded. The results presented here refer to the modeling using zero-inflated and non-zero-inflated negative binomial probability distributions. Table 1 presents the deviance information criteria (DIC) for these models. For SPW, the DIC values for the intercept and random-effects models were close in both probability distributions. For the models with covariates, the lowest DIC was the one with a non-zero inflated negative binomial distribution. For CS, the intercept and random-effects model with the lowest DIC was the one with non-zero inflated negative binomial distribution and the DIC of the covariate models were close. From these results, we considered the non-zero inflated negative binomial probability distribution for the SPW and CS models.

**Table 1:** Deviance information criteria (DIC) values for models considering only intercept and random effects and models with the addition of covariates, with negative binomial probability distribution inflated and non-inflated of zeros, for syphilis in pregnant women and congenital syphilis in the municipalities of the state of São Paulo, 2007 to 2018.

Disease	Negative binomial probability distribution	Intercept and random effects	Intercept, random effects and covariates
Syphilis in pregnant women	Non-inflated of zeros	22,200	22,134
	Zero-inflated	22,198	22,149
Congenital syphilis	Non-inflated of zeros	15,921	15,907
	Zero-inflated	15,937	15,905

**Figure 1:**
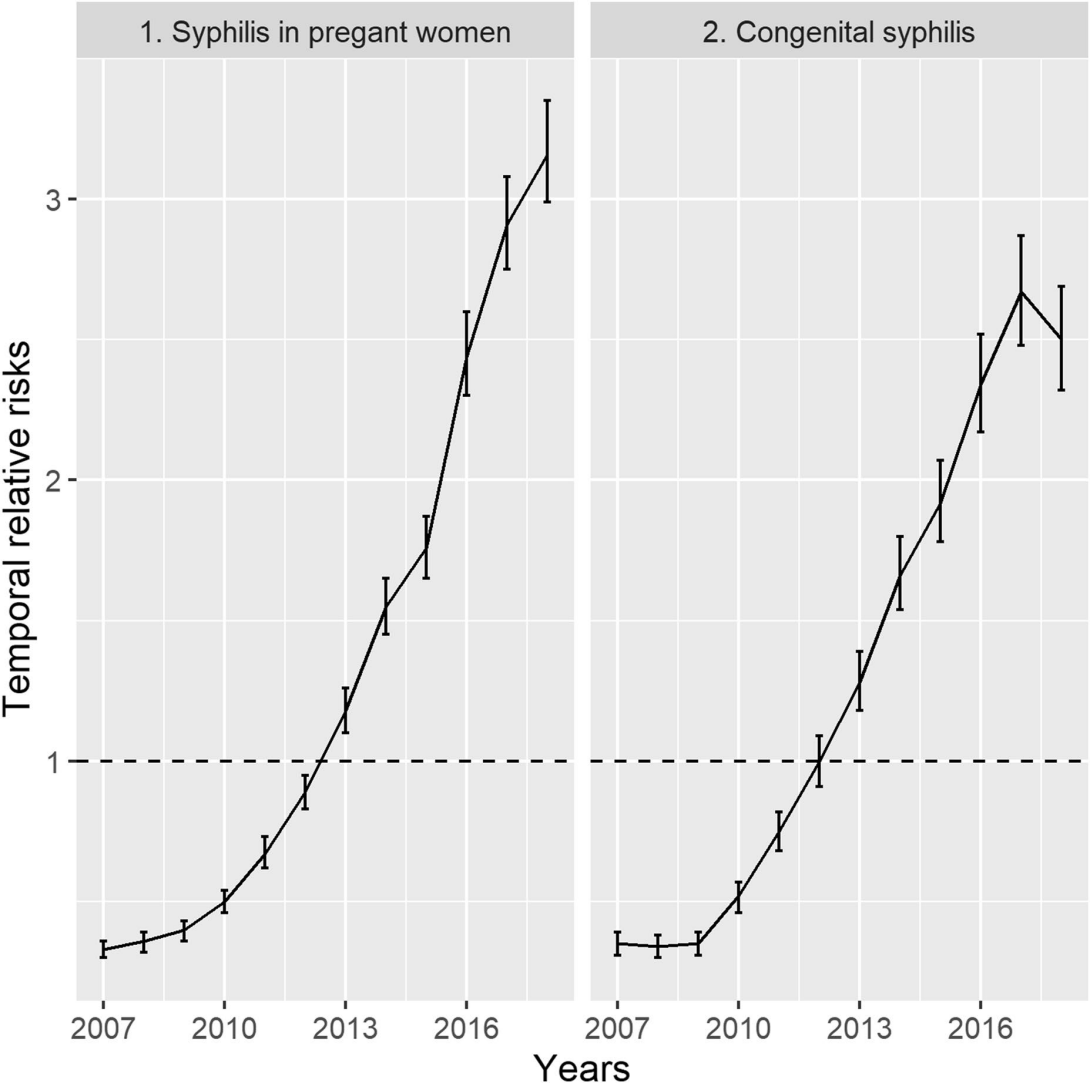
Posterior mean temporal relative risks and 95% credible intervals for the combined, unstructured and structured temporal random effects for the occurrence of syphilis in pregnant women and congenital syphilis per year, 2007 to 2018, state of São Paulo.

We commenced with the intercept and random-effects models for both diseases, presenting first the temporal relative risks (RR), spatial RR, and predicted RR, and then the results of the models with the inclusion of covariates. Figure 1 shows the temporal RR for SPW and CS, which presented a large increase during the study period. The temporal RR of SPW increased 8.6 times between 2007 and 2018 (from 0.33 to 3.16), presenting significant results between the years. Between 2007 and 2017, there was an increase in the temporal RR of CS (although it was slightly less pronounced), while there was a decrease in 2018. Between 2007 and 2017, the RR increased 6.6 times (0.35 to 2.67) and the RR of 2018 (2.50), although not significant in relation to 2017, may have represented a reversal of the temporal trend of the increasing incidence of CS.

**Figure 2:**
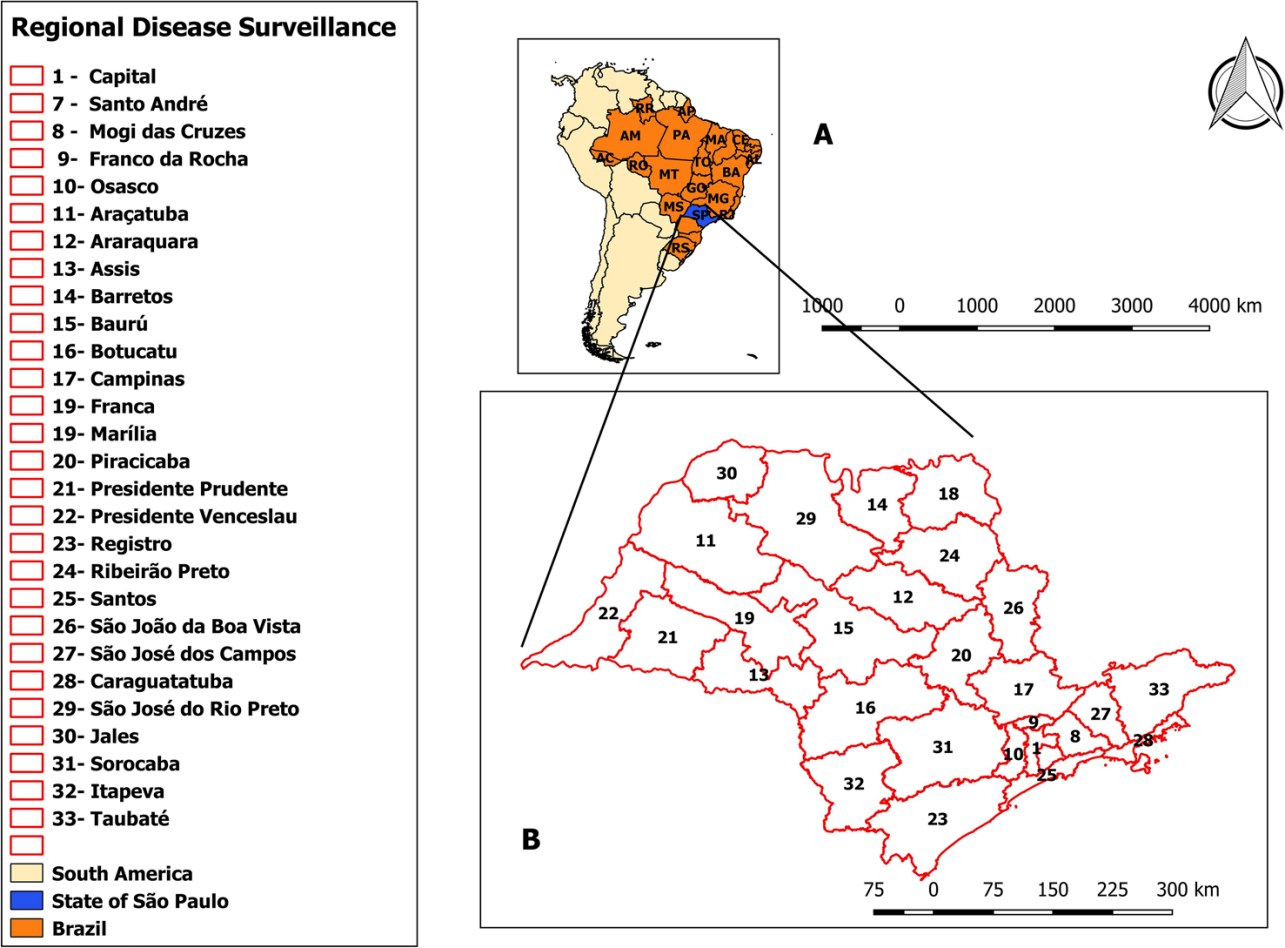
Geographic location of the study area: (**A**) Location of the state of São Paulo in relation to Brazil and South America; (**B**) Location of regional disease surveillance areas in the state of São Paulo. Maps created using the Free and Open Source QGIS version 3.16.4 (https://qgis.org).

**Figure 3:**
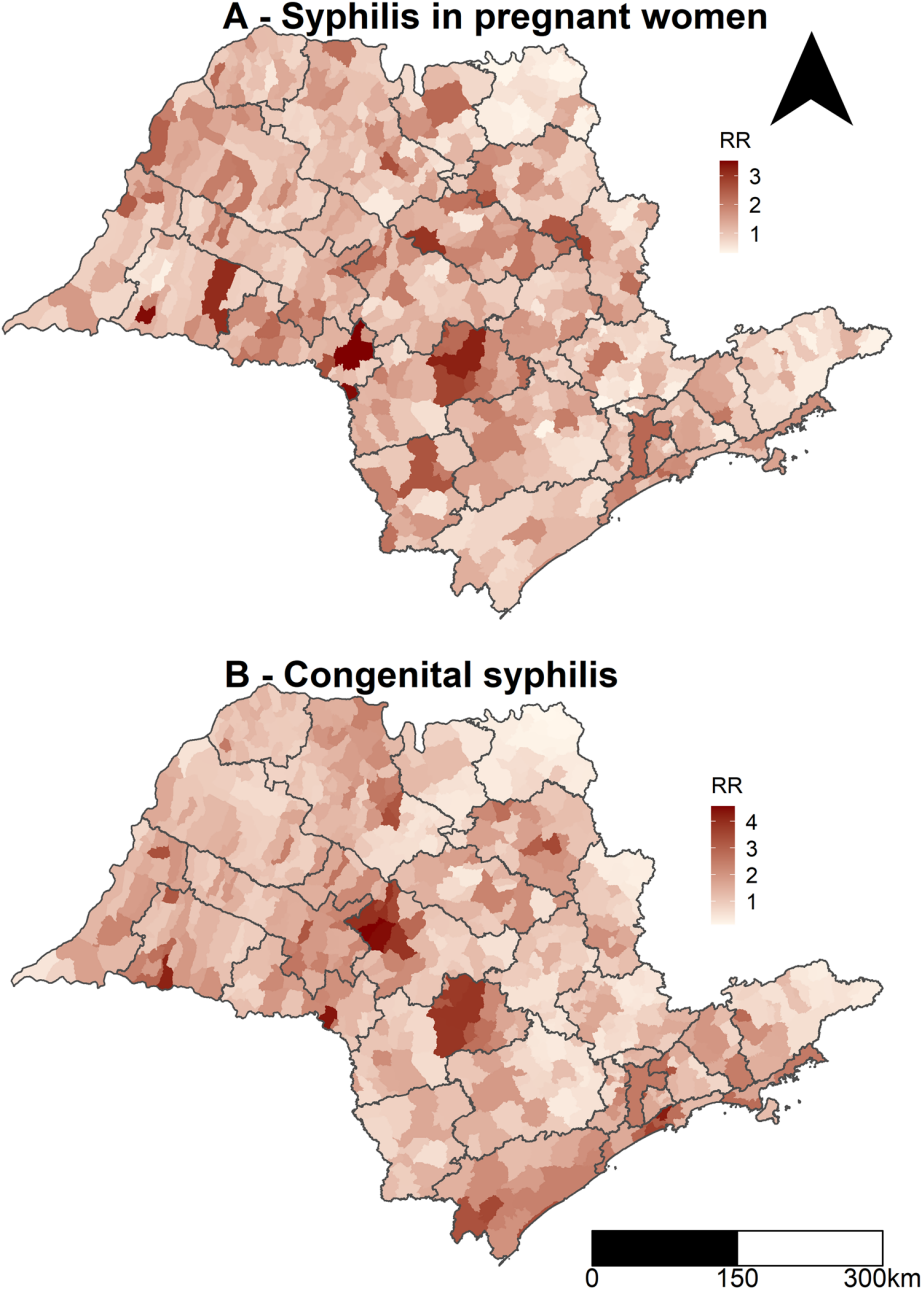
Posterior mean spatial relative risks for the combined, unstructured and structured spatial random effects for the occurrence of syphilis in pregnant women (**A**) and congenital syphilis (**B**) per municipality and regional disease surveillance area, 2007 to 2018, state of São Paulo. Maps created using the Free and Open Source R Program version 3.6.1 (https://www.r-project.org/).

Figure 2 shows the SP map divided into 28 regional disease surveillance areas (RDSs), while Fig. 3 shows the spatial RR for SPW and CS for the municipalities and RDSs for the whole period with the global rates for both diseases. The spatial RR for SPW ranged from 0.33 (municipality of Pedregulho in RDS 13) to 3.52 (Santa Cruz do Rio Pardo in RDS 8). The spatial RR for CS ranged from 0.13 (Igarapava in RDS 13) to 4.57 (Presidente Alves in RDS 10).The RDSs with municipalities with higher risks of SPW were São Paulo (RDS 1), Itapeva (RDS 27), Botucatu (RDS 16), Assis (RDS 8), Araraquara (RDS 6), and Presidente Prudente (RDS 16). In relation to CS, the following areas had municipalities with higher risks: São Paulo (RDS 1), Santos (RDS 25), Registro (RDS 18), Botucatu (RDS 16), Bauru (RDS 15), Assis (RDS 8), and Presidente Prudente (RDS 21).

**Figure 4:**
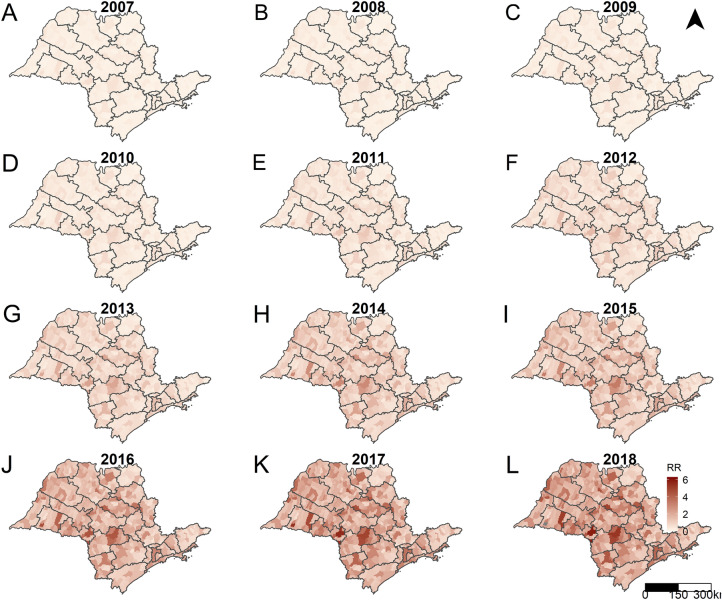
Posterior mean relative risks for syphilis in pregnant women per year, municipalities and regional disease surveillance areas, state of São Paulo, 2007 (A) to 2018 (L). Maps created using the Free and Open Source R Program version 3.6.1 (https://www.r-project.org/).

**Figure 5:**
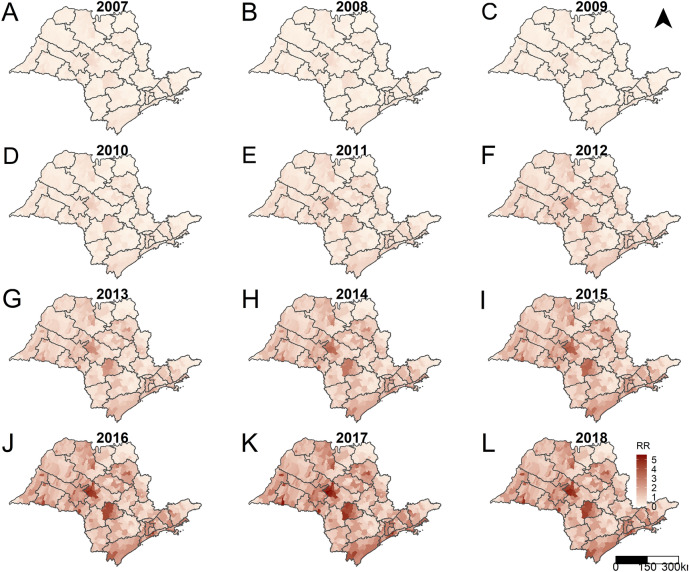
Posterior mean relative risks for congenital syphilis per year, municipalities and regional disease surveillance areas, state of São Paulo, 2007 (A) to 2018 (L). Maps created using the Free and Open Source R Program version 3.6.1 (https://www.r-project.org/).

Figures 4 and 5 present the predicted RR for SPW and CS for the years of the study period, municipalities, and the RDSs of SP. We obtained these values through the intercept random-effects models considering the spatial and temporal autocorrelations and the interactions between them; therefore, the predicted RR were adjusted taking these into account. On comparison, the gross RR for SPW ranged from 0 to 20.84, while the adjusted RR ranged from 0.05 to 6.31. Similarly, with CS, the gross RR ranged from 0 to 33.33, and the adjusted RR ranged from 0.02 to 5.44.

**Figure 6:**
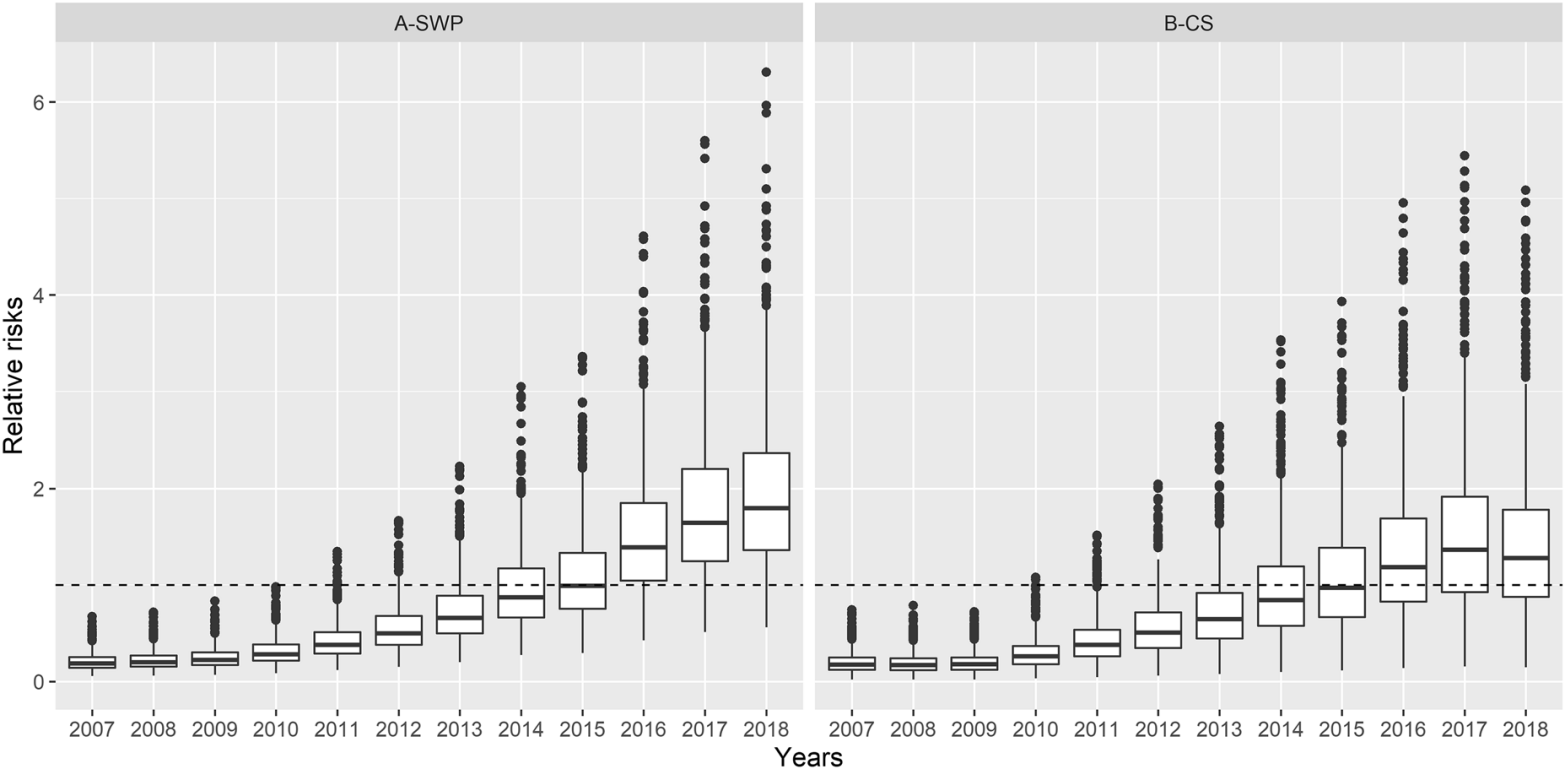
Boxplots with the posterior mean relative risks for syphilis in pregnant women (A-SWP) and congenital syphilis (B-CS) per year, municipalities of the state of São Paulo, 2007 to 2018.

Figure 6, which presents the predicted RR for both diseases per year using box plots indicates the dimension of the increase in the occurrence of SPW and CS. The predicted values of the RR for SPW and CS showed continuous growth over the years (Fig. 1). Furthermore, this growth was widespread, occurring in all the municipalities of SP. The RR presented values below the unity for all the municipalities until 2009 and 2010, for CS and SPW, respectively. In 2015, approximately 50% of the municipalities of SP presented an RR greater than or equal to unity. In 2017 and 2018, when CS and SPW cases peaked, the proportion of municipalities with RR over unity was 71% (457 in 645) and 92% (590 in 645) for CS and SPW, respectively.

After presenting the results of the intercept plus random-effects models, we finalized the results with the inclusion of covariates in these models. The exploratory analyses of the covariates showed that the proportion of women between 18 and 24 years old with no education or an incomplete first cycle of elementary school, proportion of people between 15 and 19 years old who did not work or study, proportion of LB to adolescent mothers, proportion of mothers with fewer than four antenatal care consultations, acquired immunodeficiency syndrome (AIDS) incidence rate, supplementary health care coverage, number of inhabitants, and demographic density presented outlier values. To address this issue, we transformed the first six covariates using the square root and the last two using the logarithm. The collinearity analysis with the variance inflation factor (VIF) revealed the necessity of removing the covariates average household income per capita, proportions of people with incomes below half the minimum wage, and demographic density.

Table 2 presents the posterior mean RR and 95% credible intervals for the covariates considered to be in the negative binomial models for SPW and CS. We considered a spatio-temporal architecture; therefore, the results for the covariates were adjusted for spatial and temporal autocorrelations and the interactions between them; the items of significance are highlighted in bold in Table 2. The model for SPW showed that the variation of 1 standard deviation (SD) of the square root of the proportion of LB to adolescent mothers, the logarithm of the number of inhabitants, and the square root of the AIDS incidence rate can result in an increase of 6%, 32%, and 9% in the risk of the occurrence of this disease, respectively. For CS, the variation of 1 SD of the proportion of pregnant women with inadequate antenatal care, the square root of the proportion of mothers with less than four antenatal care consultations, the logarithm of the number of inhabitants, and the square root of the AIDS incidence rate would result in an increased risk of CS by 11%, 7%, 22%, and 9%, respectively. We also found significant results for the interaction between the logarithm of the number of inhabitants and the square root of the AIDS incidence rate, for both SPW and CS. Supplementary Material 3 presents the descriptive analysis of the covariates considered in the models.

**Table 2:** Posterior mean relatives risks (RR) and the 95% credible intervals (95% CI) for the covariates considered in the negative binomial models for syphilis in pregnant women (SPW) and for congenital syphilis (CS), state of São Paulo, 2007 to 2018. Cells in bold represent that the 95% CI for the RR did not include the unit.

Covariate	SPW		CS	
	RR	95% CI	RR	95% CI
Intercept	0.48	0.45–0.50	0.40	0.37–0.45
Proportion of women of childbearing age	1.02	0.97–1.07	1.01	0.95–1.07
Proportion of women between 18 and 24 years old with no education or with incomplete 1st cycle of elementary school*	0.98	0.92–1.03	0.94	0.87–1.02
Gini index	1.00	0.93–1.06	0.94	0.87–1.02
Proportion of people with an income below ¼ minimum wage	1.02	0.94–1.11	1.11	0.99–1.23
Proportion of people between 15 and 19 years old who did not work or study*	1.00	0.92–1.08	1.07	0.97–1.18
Proportion of people between 15 and 19 years old who did not attend school	1.04	0.96–1.12	0.95	0.86–1.04
Proportion of pregnant women with inadequate antenatal care	1.03	0.97–1.09	**1.12**	**1.04–1.20**
Proportion of live births to adolescent mothers*	**1.06**	**1.00–1.11**	1.05	0.98–1.13
Proportion of mothers with less than four antenatal care consultations*	1.00	0.96–1.04	**1.07**	**1.01–1.13**
Number of inhabitants**	**1.32**	**1.21–1.44**	**1.22**	**1.09–1.38**
Presence of prison in the municipality (yes)	0.99	0.85–1.16	1.13	0.93–1.35
Municipality that performed deliveries (yes)	1.00	0.98–1.01	0.97	0.86–1.05
AIDS incidence rate*	**1.09**	**1.05–1.14**	**1.09**	**1.04–1.15**
Primary health care coverage	1.01	0.96–1.05	1.00	0.94–1.06
Supplementary health plan coverage*	0.96	0.89–1.04	1.03	0.94–1.14
Interaction: Number of inhabitants** and AIDS incidence rate*	**1.07**	**1.02–1.12**	**1.09**	**1.02–1.15**

*Transformed by the square root.

**Transformed by the logarithm.

## Discussion

This study showed a continuous increase in the temporal RR of SPW from 2007 to 2018, and CS, from 2007 to 2017. Over these years, the incidence of SPW and CS increased 8.6 and 6.6 times, respectively. Moreover, this increase occurred en bloc in approximately all the municipalities of SP. This occurred in the richest and most populated state of Brazil. SP represents more than 31% of the Brazilian gross domestic product, with approximately 46 million inhabitants, and is ranked the third most populous and cosmopolitan political unit in South America^15^.

In the analyses of the socioeconomic and demographic conditions, link to health services, and presence of transmissible comorbidities such as AIDS, which also indirectly involved aspects related to programmatic vulnerability, we observed that the increase in SPW was influenced by teenage pregnancy, municipalities with a large number of inhabitants, and AIDS incidence. The conditions that influenced the increase of CS were municipalities with a large number of inhabitants, incomplete antenatal care, and AIDS incidence. We also found a significant interaction between the number of inhabitants and the AIDS incidence rate for both SPW and CS.

This study had limitations and issues of concern. We used secondary data with the possibility of underreported cases. Another limitation was the inability to consider variables indicative of programmatic vulnerability, such as penicillin availability in PHC and testing coverage for syphilis, due to the lack of free access to this type of data. This information would have facilitated our discussion on aspects that could be related to the persistence of congenital syphilis. Issues of concern included the presence of temporal and spatial correlation in our response variables, the existence of numerous municipalities with small populations, and small numbers of LB generating random fluctuations in SPW and CS rates and RR.

Part of these deficiencies in the data were overcome by the modeling framework we used. We developed our study with a space–time architecture that accounted for the spatial and temporal dependence. This aspect was considered through the use of Bayesian latent Gaussian models, so that the results obtained were adjusted for the spatial and temporal autocorrelations and the interactions between them^9,10^. Since space and time were part of the modeling, the Bayesian inference allowed us to obtain the RR describing the behavior of the diseases studied according to time (years), space (municipalities), and space–time (municipalities according to years). These last two results, related to the mapping of diseases and their risks, are invaluable tools in view of the possibility of knowledge of areas with higher risks, assisting in health decision-making, interpreting the implementation of existing actions, and intervening to reduce injuries through prevention programs^8^. Moreover, the consideration of neighborhood relationships in the models allowed the generation of smooth RR for the municipalities, avoiding random fluctuations that could be present in those with reduced numbers of LB.

The ecological design we used could be considered as a weakness of our study as we did not evaluate the response variable at the individual level, but aggregated on space and time. On the other hand, it is also a strength, considering that ecological studies set the stage for examining causal mechanisms and provide a better approach for exploring exposures that are easier to measure in groups than at the individual level^16^. Furthermore, inferences were uniformly made for aggregates and not individuals to avoid ecological fallacy. We interpreted our results based on the characteristics of the regions and not individually in relation to pregnant women and LB.

Our results showed a continuous increase in the incidence of CS and SPW. Since these two diseases display a synergistic behavior, an increase in the second^5^ can lead to management difficulties in the first^17^. This demonstrated the importance of studies on this dynamic. An analysis of the global load of SPW and CS showed that Europe was the only region that presented a level close to elimination, despite estimates of declines in Southeast Asia. The Eastern Mediterranean and American regions showed the largest estimated increases in the prevalence of SPW^5^. A study in Mexico showed that only 53% of pregnant women underwent diagnostic tests^18^ and recently, a study conducted between 2012 and 2016 in the United States, demonstrated a significant increase in CS, from 8.4 to 15.7 cases/100,000-LB^19^.

This incidence can be explained by the expansion of the coverage of tests to detect diseases and the improvement in surveillance^20^. In Brazil, the availability of a rapid test for syphilis increased from 31,500 in 2011 to 3,156,410 in 2014^21^. In SP, this increase was greater: from 1000 tests being available in 2011 to 1,314,700 in 2017^22^. Such progress was also probably due to funds from the Unified Health System (Sistema Único de Saúde—SUS) under the “*Rede Cegonha*” program, which aims toward the improving care for women during pregnancy, childbirth, and postpartum, for newborns and children up to 2 years old^23^, and investing in the mother and child binomial^24^. A study conducted in Brazil confirmed this tendency, showing that the increase in the capacity to identify asymptomatic cases of syphilis resulted in an increase in the notifications of acquired, gestational, and congenital syphilis^21^. It is important to highlight that the identification of cases through proper testing must target all groups vulnerable to this health problem.

A recent study^5^ pointed out that the increase in SPW and CS is closely related to the expressive increase in AS rates in the general population and people socially vulnerable to human immunodeficienvy virus (HIV) and sexually transmitted infections (STI), such as men who have unprotected sex with men; and those not covered by the search actions, such as those in the antenatal care protocol. Furthermore, researches^14,25,26^ demonstrated the importance of adequate antenatal care, syphilis screening, and correct and timely treatment for the prevention of syphilis. For Kimball et al.^26^, late seroconversion and the lack of early identification of cases were reflections of the increase in groups at risk and issues of social vulnerabilities such as the lack of adequate housing, education, and access to information.

The increase in SPW and CS incidence was also influenced by the decreased use of condoms and loss of opportunity for treatment when penicillin was not administered in PHC, as well as the global crisis supply shortages between 2014 and 2016^11^. This evidence was consistent with the results of this research, which showed a positive association between SPW and the proportion of LB to adolescent mothers, municipalities with a large number of inhabitants, and the incidence rate of AIDS. The evidence was also consistent with the results obtained for CS, which showed a positive association with the proportion of pregnant women with inadequate antenatal care, proportion of mothers with fewer than four antenatal care consultations, municipalities with a large number of inhabitants, and AIDS incidence rate. Some of the variables associated with CS were also associated with SPW, highlighting the finding of a previous study that the control of CS is directly linked to the management of SPW during antenatal care^25^. Another point to be highlighted is the interaction between the number of inhabitants and the AIDS incidence rate for both SPW and CS, which shows that larger municipalities with higher AIDS incidence experience a syndemic effect.

Other factors that influenced the spread of syphilis were related to the accelerated urbanization process seen in recent decades in Brazil. Consequently, there is increased social exclusion, generating segregated populations, difficulties in accessing urban services and infrastructure, greater exposure to violence, racial discrimination, and discrimination against women and children^27^. These situations, among others, affect the way a person falls ill and the health needs of a population^28^. Nevertheless, in terms of health, larger populations can provide the impetus for a municipality to present a more comprehensive care network with greater investments due to greater demand^29^. However, municipalities with larger population sizes present greater disparities in their social determinants of health, including access to health services^28^.

SP, with more than 20% of the Brazilian population, is a good example of this dichotomy. On one hand, 32.5% of municipalities in SP have been classified as dynamic, with high wealth and good levels of social indicators (longevity and education); while on the other, 43.9% of its municipalities demonstrate inequalities, with high levels of wealth, but with unsatisfactory social indicators^30^. People are diverse in terms of culture and education, and they move indistinctly from the capital to the interior or vice versa^31^.

The intervening factors in the current scenario of SPW and CS are probably related to access and capacity of prenatal care. The Ministry of Health provides the evaluation of health teams and care offered to users, as implemented in the National Program for Improving Access and Quality of Primary Care in the Family Health Strategy^32^. In SP, 90% of the teams participated in the last evaluation cycle, and 84% of the units applied intramuscular benzathine penicillin administration. Nonetheless, in spite of the many obstacles, Brazilian PHCs contributed to the control of syphilis. In 2019, of the 12,650 cases of SPW registered in SP, 4013 evolved to CS, and approximately 72% of CS cases were avoided. Of those who progressed to CS, 28% had maternal reinfections close to delivery even after adequate treatment, including those who changed partners or had untreated partners, and 14% were diagnosed with syphilis only in the third trimester of pregnancy^33^.

Major challenges that need to be overcome on a worldwide scale are the issues related to the incorrect treatment of syphilis during pregnancy and the lack of audits to identify flaws in the care process and the adequacy of the qualifications of health professionals. A study conducted in Brazil demonstrated these difficulties^32^ and another one conducted in Argentina pointed out that, of the children identified with CS and born in hospitals (corresponding to 96% of the total CS cases), only 47% of their mothers completed the antenatal care serological screening, and only four were adequately treated^34^.

The lack of knowledge and the importance of health monitoring during pregnancy have been associated with populations of greater social vulnerability, low education, and non-white skin color. Women with low education have less access to information, which limits their knowledge of health care, particularly on the prevention of sexually transmitted diseases such as syphilis infection^35,36^. Equally, it is worth mentioning the existence of more vulnerable population groups in each municipality, especially with regard to adolescents, as evidenced by the results. A national study showed the association of so-called young people “*nemnem*” or NEET (Not in Education, Employment or Training) to low parental education, lower income, and rural areas^37^. A study in the United Kingdom indicated that young people were more likely to become unemployed, use drugs and alcohol, be involved in crimes, have poor health, and become pregnant in adolescence^38^.

Regarding the association of both situations of the disease (SPW and CS) with the AIDS incidence rate, HIV and syphilis affect populations with similar profiles in which co-infection is common. According to the literature, people infected with other STI, including syphilis, are three to five times more likely to acquire HIV^39^. Moreover, the prevalence of syphilis also occurs more frequently among HIV-positive pregnant women than among HIV-negative women, because pregnant women living with HIV may have a weaker immune systems than other pregnant women^40^. Moreover, the effect of AIDS and HIV on the occurrence of SPW and CS is enhanced in municipalities with large populations.

Considering that this study followed all the precepts of ecological design, it is necessary to indicate the need for research that can analyze, as robustly as this study, issues directly related to individual characteristics and can contribute to the evaluation of the persistence of SPW and CS, as well as the facilitation of qualified audits to assess programmatic vulnerabilities involving their management, care and prevention. Although Brazil has an extensive range of technical materials and well-established clinical protocols that regulate the management of SPW and CS, it is still necessary to standardize the therapeutic conduct of health professionals and identify treatments with alternative drugs that can be used in periods of worldwide shortages of penicillin, among other factors.

The results obtained in this study may also be useful in the design of new studies for the evaluation of SPW and CS surveillance and control in SP, both from a spatial and temporal point of view. The en bloc increase in the occurrence of both diseases indicates that actions are required in almost all the municipalities of SP. However, the identification of municipalities and RDSs at the greatest risk for these diseases, in addition to the identification of their characteristics, also points to priority areas for the development and implementation of these actions. If, on one hand, the growing tendency of SPW points to difficulties in its control, the reversal of the temporal tendency of CS, even if not statistically significant, can be an indication of the resoluteness of the actions that have been developed. It is also important to highlight that these actions to control SPW and CS strengthen the strategic surveillance systems and cooperate with the sustainable development goals.

## Methods

### Type, period, area, and study population

This ecological study was based on secondary data with spatial and temporal components. The study area comprised 645 municipalities and 28 RDSs in SP (Fig. 2), and the study period was from 2007 to 2018. SP occupies an area of 248,220 km^2^ and in 2018 it had a population of 43,993,189 inhabitants. Based on the 2010 population census, the human development index for SP was 0.783. Between 2007 and 2018, infant mortality rates and the proportions of pregnant women who had more than six consultations evolved from 13.1 to 10.8 deaths aged under one year per 1000 LB and from 74.8 to 79.7%, respectively^41^. The study population included cases of SPW and CS in LB in SP for which the SINAN received notifications, from 2007 to 2018.

### Source of data and variables

The notified cases of SPW and CS, presented by municipality and year, constituted the two response variables in this study. We obtained this data from the Epidemiological Bulletin of SP^7^, which is prepared and disseminated periodically by the STI and HIV/AIDS Program of SP. Information on the year and municipality of LB were obtained from the Live Birth Information System^41^. We also used the data obtained from the SINAN to characterize women with gestational syphilis and congenital syphilis.

Possible factors associated with the occurrence of the conditions studied were socioeconomic, demographic, and health care variables. We considered the proxies for the socioeconomic and demographic conditions the following variables obtained for each of the municipalities: proportion of women of childbearing age (15 to 49 years); proportion of women between 18 and 24 years of age with no education or with an incomplete first cycle of elementary school; average household income per capita in Reais (the Brazilian currency); Gini index; proportion of people with an income below a quarter and half of the minimum wage; proportion of people between 15 and 19 years of age who did not work or study; proportion of people between 15 and 19 years of age who did not attend school; number of inhabitants; and demographic density. This information was obtained from the 2010 Population Census database provided by the Brazilian Institute of Geography and Statistics^42^. In this group, we also considered the presence of prisons in the municipality. It was a categorical variable with a unique value (yes or no) for each municipality during the entire study period.

We considered the following proxies for the factors related to health care: the proportion of pregnant women with inadequate antenatal care, proportion of LB to adolescent mothers (aged 19 years or less), municipality that performed deliveries (categorical: yes or no), and proportion of mothers with less than four antenatal care consultations. This information was obtained from the Live Birth Information System^41^. For the construction of the variable proportion of pregnant women with inadequate antenatal care we considered information for the year 2014, which was the median year period and the first year for which information was available. In the calculation, the number of pregnant women with inadequate antenatal care (women who began antenatal care after the first trimester of pregnancy and those who, although they had started antenatal care until the third month of gestation, had less than three consultations) was uses as the numerator, and the number of LB as the denominator. The variable proportion of LB to adolescent mothers, municipality that performed deliveries, and proportion of mothers with less than four antenatal care consultations were obtained for each year of the study period and for each municipality.

We also considered, as part of the conditions related to healthcare, the AIDS incidence rate and the primary and supplementary health care coverages. The AIDS incidence was calculated by dividing the number of notified AIDS cases by the population (cases per 100,000 inhabitants) for each municipality and year of the study period. The data of notified AIDS cases were obtained from the Epidemiological Bulletin of SP^5^. Primary and supplementary health care coverages for each municipality and year were obtained from the e-Gestor AB website^43^ and the ANS Tabnet website^44^, respectively.

Based on the information presented, a database was constructed containing the numbers of cases of SPW and CS in each municipality and for each year, and the expected cases of the two diseases by municipality and year. These were calculated from the global rates of detection of SPW and the incidence of CS (per thousand LB-years) for the entire study period. The expected values of SPW and CS were obtained for each year and municipality by multiplying the global rates by the number of LB and dividing the result by 1000. These values represented the expected number of SPW and CS cases in a specified municipality in a given year, if the city had the same rates as the entire study area. To this database, socioeconomic, demographic, and health care variables (presented above) were added and were considered as covariates. This database is available in Supplementary Material 2.

### Data analysis

We performed a descriptive analysis to characterize SPW and CS cases considering the socioeconomic and access variables for the first (2007) and the last (2018) year of our study period. For SPW, we evaluated age, skin color, education, pregnancy trimester at the time of diagnosis, and treatment information. For CS, we evaluated maternal characteristics, such as age, skin color, education, prenatal care, time of diagnosis, treatment, and partner's treatment.

An exploratory analysis was conducted to assess the existence of outliers and quantities of zeros in the response variables, identify collinearity between the covariates, and evaluate relationships between the two outcomes and each of the covariates. The collinearity analysis was performed by calculating VIF. Covariates with VIF > 3 were considered to be collinear with one or more of the others and were not included in the modeling^45^.

The modeling took into account a space–time architecture; therefore, random spatial and temporal random effects and those relative to the interaction between space and time, were considered. The spatial random effects were modeled according to the model proposed by Besag et al. (1991)^46^ and called the Besag-York-Molié (BYM). It consisted of two types of random effects: a structured spatial random effect that represented local spatial dependence between municipalities, and an unstructured spatial random effect that represented global spatial dependence^46^. Here, the BYM2 model was used according to the modifications proposed by Riebler et al. (2016)^47^. The contiguity was considered as a criterion to establish the neighborhood matrix between municipalities. The time dependence was modeled considering an unstructured random-effects and a structured one, which was named the random walk type 1 (RW1). The interaction between space and time was modeled by two unstructured random effects, one in space and another in time^9^. The mathematical notations of these models are presented in Supplementary Material 4.

We ran the models using the integrated nested Laplace approximation (INLA) approach in a Bayesian context^48^. Initially, for both SPW and CS models, the Poisson probability distributions, uninflated zeros, and inflated zeros were considered. Since these models showed an overdispersion (variances greater than the respective averages), we used negative binomial distributions, non-inflated zeros and inflated zeros in the modeling. The expected cases of SPW and CS were considered as offsets in the modeling; therefore, the results obtained were interpreted on a natural scale as RR to the global rates of detection of SPW and incidence of CS. Initially, only the intercept and random effects models were examined, and later, the models with covariates were added. In the modeling, these were first centered by subtracting the respective averages and then scaled by dividing the values obtained by the respective SDs. We also investigated possible significant interactions among the covariates.

The priors adopted for the random effects were those recommended by Simpson et al. (2017)^49^, that is, priors with a penalized complexity. Non-informative priors were adopted for the fixed effects models. The DIC was used as a measure of the degree of adjustment of the models, so that the lower the DIC, the better was the model adjustment^9^. We conducted the analysis using the R program version 3.6.1^50^ and the R-INLA^48^, INLAOutputs^51^, devtools^52^, tidyverse^53^, sf^54^, spdep^55^ and lattice^56^ packages. The codes we used to run our models are presented in the Supplementary Material 5.

### Ethical considerations

All information used in this study was obtained from sources with universal public access and, therefore it was not submitted to a research ethics committee for approval. All methods we used to analyze our data were carried out in accordance with relevant guidelines and regulations.

## Supplementary Information

Supplementary Information 1.

Supplementary Information 2.

Supplementary Information 3.

Supplementary Information 4.

Supplementary Information 5.
