# Supplementary material for: Spatiotemporal dynamics of syphilis in pregnant women and congenital syphilis in the state of São Paulo, Brazil
Source: Sci Rep. 2022 Jan 12;12:585. doi: 10.1038/s41598-021-04530-y (PMC8755837; doi:10.1038/s41598-021-04530-y)
Supplement: Supplementary file 1 — Supplementary Information 1. [file 41598_2021_4530_MOESM1_ESM.pdf]

Supplementary Material 1

Table 1 - Characteristics of pregnant women notified with syphilis and number of municipalities with adequate treatment for syphilis in pregnancy, state of São Paulo, Brazil, 2007 and 2018

Characteristics	2007 (n=1,072)	2018 (n=12,637)
	N(%)	N(%)
**Age (years)**		
≤ 19	162 (15.1)	2.988 (23.6)
20 to 29	542 (50.6)	6.870 (54.4)
30 to 39	324 (30.2)	2.506 (19.8)
40 and more	44 (4.1)	273 (2.2)
**Skin color**		
White	555 (51.8)	5,361 (42.4)
Black	131 (12.2)	1,367 (10.8)
Yellow	6 (0.6)	82 (0.6)
Brown	305 (28.5)	5,004 (39.6)
Indigenous	12 (1.1)	21 (0.2)
NA	63 (5.9)	802 (6.3)
**Education (years)**		
Without education	12(1,1)	19(0,2)
1 to 4	193(18,0)	671(5,3)
5 to 9	418(39,0)	3.073(24,3)
10 to 12	224(20,9)	5.452(43,1)
>12	15(1,4)	391(3,1)
NA	210(19,6)	3.031(24,0)
**Trimester of pregnancy at diagnosis**		
1º trimester	352 (32.8)	6,555 (51.9)
2º trimester	348 (32.5)	3,056 (24.2)
3º trimesteer	274 (25.6)	2,606 (20.6)
NA	98 (9.1)	420 (3.3)
**Type of treatment performed**		
Adequate	872 (81.3)	11,061 (87.5)
Inadequate	200 (18.7)	1,576 (12.5)
**Municipalities with adequate treatment for syphilis in pregnancy***		
>80% with adequate treatment	110 (60.4)	306 (66.4)
51 to 80% with adequate treatment	26 (14.3)	88 (19.1)
≤50% with adequate treatment	17 (9.3)	34 (7.4)
Without adequate treatment	29 (15.9)	33 (7.2)
Total de municípios	182	461

Source: Information System for Notifiable Diseases (SINAN)

* Number of municipalities

Table 2 – Maternal characteristics of congenital syphilis cases, state of São Paulo, Brazil, 2007 and 2018

Maternal characteristics	2007 (n=783)	2018 (n=4.011)
	N(%)	N(%)
**Age (years)**		
≤ 19	84 (10.7)	841 (21.0)
20 to 29	399 (51.0)	2,163 (53.9)
30 to 39	241 (30.8)	828 (20.6)
40 and more	48 (6.1)	94 (2.3)
NA	11 (1.4)	85 (2.1)
**Raça/cor**		
White	353 (45.1)	1,615 (40.3)
Blck	61 (7.8)	294 (7.3)
Yellow	9 (1.1)	6 (0.1)
Brown	172 (22.0)	1,750 (43.6)
Indigenous	10 (1.3)	3 (0.1)
NA	178 (22.7)	343 (8.6)
**Education (years)**		
Without education	14 (1.8)	10 (0.2)
1 to 4	117 (14.9)	167 (4.2)
5 to 9	291 (37.2)	976 (24.3)
10 to 12	129 (16.5)	1,491 (37.2)
>12	9 (1.1)	100 (2.5)
NA	223 (28.5)	1,267 (31.6)
**Antenatal care**		
Yes	642 (82.0)	3,231 (80.6)
No	121 (15.5)	641 (16.0)
NA	20 (2.6)	139 (3.5)
**Moment of maternal diagnosis**		
During the antenal care	400 (51.1)	2,287 (57.0)
In delivery/curettage	329 (42.0)	1,490 (37.1)
After delivery	14 (1.8)	104 (2.6)
Not performed	4 (0.5)	27 (0.7)
NA	36 (4.6)	103 (2.6)
**Maternal treatment**		
Adequate	19 (2.4)	287 (7.2)
Inadequate	452 (57.7)	1,966 (49.0)
Not performed	252 (32.2)	1,297 (32.3)
NA	60 (7.7)	461 (11.5)
**Partner’s treatment**		
Yes	77 (9.8)	926 (23.1)
No	577 (73.7)	2,263 (56.4)
NA	129 (16.5)	822 (20.5)

Source: Source: Information System for Notifiable Diseases (SINAN)
