# Supplementary material for: Spatiotemporal dynamics of syphilis in pregnant women and congenital syphilis in the state of São Paulo, Brazil
Source: Sci Rep. 2022 Jan 12;12:585. doi: 10.1038/s41598-021-04530-y (PMC8755837; doi:10.1038/s41598-021-04530-y)
Supplement: Supplementary file 3 — Supplementary Information 3. [file 41598_2021_4530_MOESM3_ESM.pdf]

**Supplementary Material 3 – Descriptive analysis of the covariates considered in the negative binomial models for syphilis in pregnant women and congenital syphilis, state of São Paulo, 2007 to 2018**.

| **Covariates** | **Mean** | **Standard deviation** | **Mini-mum** | **1º quartil** | **Median** | **3º quartil** | **Maxi-mum** |
|---|---|---|---|---|---|---|---|
| **Continuous covariates** | | | | | | | |
| Proportion of women of childbearing age (%) | 53.66 | 2.51 | 46.02 | 52.02 | 53.69 | 55.34 | 59.69 |
| Proportion of women between 18 and 24 years old with no education or with incomplete 1st cycle of elementary school (%) | 3.51 | 2.43 | 0.00 | 1.86 | 3.23 | 4.71 | 20.85 |
| Gini index | 0.46 | 0.06 | 0.33 | 0.42 | 0.46 | 0.49 | 0.69 |
| Proportion of people with an income below ¼ minimum wage (%) | 6.64 | 4.52 | 0.54 | 3.96 | 5.29 | 8.08 | 33.95 |
| Proportion of people between 15 and 19 years old who did not work or study (%) | 16.21 | 4.60 | 3.62 | 13.06 | 15.81 | 18.67 | 49.06 |
| Proportion of people between 15 and 19 years old who did not attend school (%) | 35.60 | 6.48 | 13.25 | 31.41 | 35.33 | 39.72 | 64.15 |
| Proportion of pregnant women with inadequate antenatal care (%) | 15.56 | 6.56 | 0.00 | 11.01 | 15.25 | 19.32 | 38.36 |
| Proportion of live births to adolescent mothers (%) | 17.65 | 5.68 | 0.00 | 13.97 | 17.20 | 20.95 | 50.00 |
| Proportion of mothers with less than four antenatal care consultations (%) | 3.77 | 3.02 | 0.00 | 1.85 | 3.33 | 5.11 | 64.88 |
| Number of inhabitants | 6.3e4 | 4.5e5 | 8.1e2 | 5.1e3 | 1.3e4 | 3.8e4 | 1.1e7 |
| Primary health care coverage (%) | 73.84 | 29.89 | 0.00 | 52.11 | 85.58 | 100.00 | 100.00 |
| Supplementary health care coverage (%) | 20.57 | 14.27 | 0.32 | 8.75 | 17.76 | 29.59 | 100.00 |
| AIDS incidence rate (per 100,000 inhab.) | 11.94 | 14.82 | 0.00 | 0.00 | 9.36 | 17.94 | 319.67 |
| **Categorical covariates** | **Proportion of yes (%)** | | | | | | |
| Presence of prison in the municipality (yes and no) | 10.70 | | | | | | |
| Municipality that performed deliveries (yes and no) | 60.47 | | | | | | |
