# Supplementary material for: Spatiotemporal dynamics of syphilis in pregnant women and congenital syphilis in the state of São Paulo, Brazil
Source: Sci Rep. 2022 Jan 12;12:585. doi: 10.1038/s41598-021-04530-y (PMC8755837; doi:10.1038/s41598-021-04530-y)
Supplement: Supplementary file 4 — Supplementary Information 4. [file 41598_2021_4530_MOESM4_ESM.pdf]

Supplementary Material 4 – The mathematical notations

1 – Syphilis in pregnant woman (SPW) models

$$y_{it} = nbinomial(\mu_{it})$$

$$\log(\mu_{it}) = \log(e_{it}) + \theta_{it}$$

$$\theta_{it} = \alpha + \sum_{1}^{n} \beta_j X_j + u_i + v_i + \gamma_t + \omega_t + \delta_{it}$$

where:

y_{it} = number of SPW cases in i = 1, 2, …, 645 municipalities and t = 1, 2,…,12 years;

e_{it} = the expected number of SPW cases in the municipality i and year t. These values were considered as offsets in our models and were obtained by indirect standardization considering the SPW global rate and the numbers of live births in each municipality and year;

Θ_{it} = the log relative risk of SPW cases in municipality i and year t, which, after appropriate exponentiation, corresponded to the SPW predicted relative risk in each municipality and year;

α = intercept;

β_j = (β_1, β_2, … β_n) corresponded to the coefficients of our covariates;

X_j = (X_1, X_2,… X_n) corresponded to our covariates

u_i = spatially structure random effect with an conditional autoregressive (CAR) structure that considers the neighborhood relationship among the municipalities; we used a Queen contiguity weight matrix to represent the municipality neighborhood relationship, which is one of the geometric matrices whose scheme is a spatially contiguous neighbors defined as two polygons that share a common boundary or vertex;

v_i = unstructured spatial random effect;

γ_t = random walk type 1 structured temporal random effect (RW1).

ω_t = unstructured temporal random effect;

δit = interaction between space and time, which was modeled by two unstructured random effects, one in space and another in time.

2 – Congenital syphilis (CS) models

$$y_{it} = nbinomial(\mu_{it})$$

$$\log(\mu_{it}) = \log(e_{it}) + \theta_{it}$$

$$\theta_{it} = \alpha + \sum_{1}^{n} \beta_j X_j + u_i + v_i + \gamma_t + \omega_t + \delta_{it}$$

where:

y_{it} = number of CS cases in i = 1, 2, …, 645 municipalities and t = 1, 2,…,12 years;

e_{it} = the expected number of CS cases in the municipality i and year t. These values were considered as offsets in our models and were obtained by indirect standardization considering the CS global rate and the numbers of live births in each municipality and year;

Θ_{it} = the log relative risk of CS cases in municipality i and year t, which, after appropriate exponentiation, corresponded to the CS predicted relative risk in each municipality and year;

α = intercept;

$\beta_j = (\beta_1, \beta_2, \ldots \beta_n)$ corresponded to the coefficients of our covariates;

$X_j = (X_1, X_2, \ldots X_n)$ corresponded to our covariates

u_i = spatially structure random effect with an conditional autoregressive (CAR) structure that considers the neighborhood relationship among the municipalities; we used a Queen contiguity weight matrix to represent the municipality neighborhood relationship, which is one of the geometric matrices whose scheme is a spatially contiguous neighbors defined as two polygons that share a common boundary or vertex;

v_i = unstructured spatial random effect;

γ_t = random walk type 1 structured temporal random effect (RW1).

ω_t = unstructured temporal random effect;

δit = interaction between space and time, which was modeled by two unstructured random effects, one in space and another in time.
